# Factors associated with pain among rural cancer survivors: Findings from an Australian longitudinal study

**DOI:** 10.1007/s00520-026-10622-0

**Published:** 2026-03-31

**Authors:** Katelyn Collins, Emma Pearse, Melanie L. Plinsinga, Alyssa Taglieri-Sclocchi, Melissa Day, Belinda Goodwin, Sonja March, Michael Ireland, Elizabeth A. Johnston

**Affiliations:** 1 Viertel Cancer Research Centre, Cancer Council Queensland, 553 Gregory Terrace, Fortitude Valley, QLD 4006 Australia; 2 School of Psychology and Wellbeing, University of Southern Queensland, Springfield, QLD Australia; 3 Australian Centre for Precision Health and Technology (PRECISE), Griffith University, Gold Coast, QLD Australia; 4 School of Psychology, University of Queensland, Brisbane, QLD Australia; 5 Department of Rehabilitation Medicine, University of Washington, Seattle, WA USA; 6 Centre for Health Research, University of Southern Queensland, Springfield, QLD Australia; 7 Centre for Epidemiology and Biostatistics, Melbourne School of Population and Global Health, University of Melbourne, Carlton, VIC Australia; 8 Manna Institute, University of Southern Queensland, Springfield, QLD Australia; 9 School of Exercise and Nutrition Sciences, Faculty of Health, Queensland University of Technology, Kelvin Grove, QLD Australia

**Keywords:** Cancer survivors, Cohort study, Pain, Quality of life, Regional and remote

## Abstract

**Purpose:**

This longitudinal study explored the prevalence and cumulative incidence of pain among rural cancer survivors, assessed the prevalence of persistent pain over time, and identified sociodemographic, clinical, and psychological factors associated with worse outcomes.

**Methods:**

This study was a secondary analysis of data from the Travelling for Treatment study. Adult cancer survivors living in Queensland, Australia, who had travelled ≥ 50 kms for cancer treatment were included (N = 659). Pain outcomes (intensity, interference, frequency) were assessed at baseline, three months, 12 months, and annually for up to five years using the Adjusted Quality of Life 8-Dimension Tool (AQoL-8D). Sociodemographic, clinical, and psychological factors were assessed at baseline. Prevalence and cumulative incidence of pain, and the proportion of cancer survivors reporting persistent pain was calculated. Cumulative link mixed models and binary logistic regression explored factors associated with pain outcomes and likelihood of experiencing persistent pain.

**Results:**

Across follow-up (median 3 years), 84% of rural cancer survivors reported moderate to unbearable pain, 64% reported serious pain at least weekly, and 66% reported that pain interfered with activities at least sometimes. Higher psychological distress was associated with worse pain intensity (aOR = 2.80, CI 2.24, 3.49), interference (aOR = 3.09, CI 2.56, 3.74) and more frequent serious pain (aOR = 2.46, CI 2.44, 2.47). Persistent pain was reported by 21% of rural cancer survivors and was also associated with increased psychological distress (aOR = 1.18, CI 2.56, 3.74).

**Conclusion:**

Pain is common among rural cancer survivors. Comprehensive interventions that address co-occurring mental and physical health comorbidities and facilitate open communication about pain are needed to ensure effective pain management and support for this population.

**Supplementary Information:**

The online version contains supplementary material available at 10.1007/s00520-026-10622-0.

## Introduction

Pain is one of the most common symptoms experienced by cancer survivors [1–3]. Cancer-related pain encapsulates any pain related to cancer [4]. It may present acutely (e.g., post-operative) and chronically (i.e., at least three months in duration), and encapsulates any form of pain including but not limited to somatic, visceral, and neuropathic pain [5]. It can impact survivors prior to, during, and for many years after, cancer treatment [6], and arises from both the disease itself (e.g., tumour growth, inflammation) and its treatment (e.g., chemotherapy, radiation, surgery; [7]. Left untreated, cancer-related pain can be significantly detrimental to quality of life, physical and cognitive function, psychological health, and even survival [6, 8]. Despite this, many cancer survivors report receiving inadequate pain management [9, 10], particularly those living in regional and remote areas (hereafter ‘rural’; [11].

Unique psychosocial factors may exacerbate the experience of pain among rural cancer survivors. For example, stoicism, defined as the endurance of hardship without emotional expression, and pre-determinism, referring to the belief that events are beyond one’s control, are common ideologies in rural communities [12, 13]. These attitudes are associated with under-reporting of pain and reduced help-seeking [14], potentially leading to worse pain outcomes. Furthermore, elevated psychological distress among cancer survivors is associated with worse pain [15, 16], which is of particular concern given that rural cancer survivors are at greater risk of mental health comorbidities compared to their metropolitan counterparts [17, 18].

Environmental barriers to effective pain management in rural areas are also well documented, such as limited availability of pain specialists, long waiting periods, poor road conditions, high cost of transportation services, and concerns regarding prescription of opioid medications [19–21]. Understanding the impact of pain, and the biopsychosocial factors associated with it, can inform efforts to improve access to pain management support and rural health outcomes. Recent evidence indicates that urban–rural disparities in pain among cancer survivors may in part be accounted for by demographic factors such as education level, income, and number of existing health conditions [22, 23]. Limited research, however, has examined the role of psychological factors and attitudes toward help-seeking, such as stoicism and pre-determinism, in this context. Furthermore, the impact and factors associated with pain among rural cancer survivors over time is currently unknown, with most of the research in this area conducted in cross-sectional, metropolitan samples [15, 24–26]. Therefore, this study aimed to: i) quantify the prevalence and cumulative incidence of pain intensity, frequency, and interference experienced by rural cancer survivors throughout the study period, ii) determine the proportion of rural cancer survivors who experience persistent pain, and iii) examine the sociodemographic, clinical, and psychological factors associated with these pain outcomes, including investigation of factors relevant to rural communities, such as stoicism and pre-determinism.

## Method

### Participants and recruitment

This study was a secondary analysis of data from a longitudinal cohort study, Travelling for Treatment (T4T), assessing patient-reported outcome measures across time among rural cancer survivors in Queensland, Australia. Findings and methodology for the T4T study have been previously described in detail [27–30]. Briefly, recruitment took place from September 2017 to June 2020, wherein adults (aged ≥ 18 years) diagnosed with cancer and staying at one of Cancer Council Queensland’s (CCQ) subsidised accommodation lodges, were invited to participate. In line with Queensland’s Patient Travel Subsidy Scheme, patients and their caregivers are eligible to stay at a CCQ lodge if travelling at least 50 kms (31 miles) to receive cancer treatment. Study invitation packs were distributed to eligible participants upon arrival by lodge staff or sent via mail to guests’ home address. Guests were contacted one week following pack distribution by a member of the research team to discuss the study invitation and answer any questions. Informed consent was obtained from all individual participants included in the study. Ethical approval for this project was obtained from the University of Southern Queensland Human Research Ethics Committee (ref. H17REA152), and all procedures were conducted in accordance with the Declaration of Helsinki.

### Data collection

Consenting participants completed paper-based self-assessment questionnaires (SAQs) at baseline, three months, 12 months, and annually thereafter for up to five years. A structured interview was also completed at baseline to gather further demographic and clinical information, such as cancer type, time since diagnosis, and comorbid health conditions. Participants were eligible for inclusion in this analysis if they i) consented to participate in the T4T study and ii) responded to the pain-related items on at least one SAQ from baseline to five years follow-up (see ‘Pain-related measures’ below).

**Sociodemographic characteristics.** Participants’ age, gender identity, and residential postcode were collected during the baseline SAQ. Residential postcode was used to generate participants’ remoteness level and area-level socioeconomic status (SES) according to the Australian Statistical Geography Standard [31] and the Socio-Economic Index for Areas [32], respectively. With the sample skewed toward lower socioeconomic advantage, SEIFA was categorised into tertiles, with the first tertile representing the most disadvantaged areas, and the third tertile representing the least.

**Clinical variables.** Participants’ clinical information, including their primary cancer type, time since diagnosis, recurrence status, and number of comorbidities were recorded during the baseline interview. Cancer type was categorised to reflect the four most common (non-skin) cancers in Australia (i.e., lung, colorectal, prostate, breast; [33] as well as an “other” category, including all other cancer types reported. Time since diagnosis was coded as a categorical variable, indicating whether participants completed the baseline interview < 1 year, 1 to < 3 years, 3 to < 5 years, or ≥ 5 years post-diagnosis. Recurrence status was assessed using a “yes” or “no” response to the question, *“Is this current (or most recent) cancer diagnosis a recurrence of a previous cancer?”*. Comorbid health conditions were assessed using the validated Charlson Comorbidity Index (CCI; [34], wherein participants were asked to specify whether they had been ever diagnosed with a specified list of common comorbidities. Responses were categorised as zero, one, or two or more comorbid health conditions.

**Psychological variables.** Measures of psychological distress, stoicism, and pre-determinism were obtained from baseline SAQs. Psychological distress over the past seven days was assessed using the validated Depression Anxiety and Stress Scale-21 (DASS-21; [35]. Mean total DASS scores were calculated and standardised to produce a global measure of distress. Data were treated as continuous, with higher scores indicating higher distress. Stoicism was assessed using the “stoicism” subscale of the Mansfield Barriers to Help-Seeking Scale [36]. Sub-scale items assessed level of agreement with a series of statements (e.g., *“I would feel better about myself knowing I did not need help from others”*) on a five-point Likert scale, where *1* = *“strongly disagree”* and *5* = *“strongly agree”*. Finally, to assess pre-determinism, the “pre-determinism” subscale of the Health Fatalism Scale [37] was applied, wherein participants rated their level of agreement with a series of statements (e.g., *“My health is determined by fate”*) on a five-point Likert scale, where *1* = *“strongly disagree”* and *5* = *“strongly agree*”.

**Pain intensity, frequency of serious pain, and pain interference*.*** Pain was assessed at each timepoint using the validated Assessment of Quality of Life-8 Dimension Tool (AQoL-8D; [38]. The pain subscale of the AQoL-8D is comprised of three items, to assess, in the past 7 days, the intensity of pain (*“How much pain or discomfort did you experience?”* where *0* = *“no pain”* and *3* = *“unbearable pain”*), frequency of serious pain (*“How often did you experience serious pain?”* (where *0* = *“very rarely”* and *4* = *“most of the time”)* and pain interference (*“How often did pain interfere with your usual activities?”* where *0* = *“never”* and *4* = *“always”*) As pain intensity, frequency of pain, and pain interference represent distinct constructs [39], each of these items were considered discrete outcomes in this analysis.

**Persistent and intermittent pain.** Participants were also allocated a score to represent changes in their pain intensity levels throughout the total study period, using the pain intensity item of the AQoL-8D. A pain intensity score of 0 on all recorded timepoints was indicative of *“no pain”,* whereas a score ≥ 1 (indicating at least moderate pain intensity) at each consecutive timepoint was considered *“persistent pain”*. Participants who alternated between *“no”* (0) and at least *“moderate”* (≥ 1) pain throughout the total study period were classified as experiencing *“intermittent”* pain.

### Data analysis

Data analysis was completed using R version 4.4.0, including the dplyr, purr, broom, and ordinal packages [40–43] (see analytic code in Supplementary File 1). Characteristics of the sample were summarised using descriptive statistics (mean, standard deviation, frequencies, percentages).

**Pain intensity, frequency of serious pain, and pain interference.** To quantify pain outcomes among rural cancer survivors, the prevalence of pain intensity, frequency of serious pain, and pain interference among the sample were described separately at each timepoint. Additionally, we report on the cumulative incidence of these pain outcomes, summarised the frequency and proportion of rural cancer survivors who reported *any* pain on the three pain items at least once during the study period. This included:

- Pain intensity: *“moderate”* to *“unbearable”*.
- Frequency of serious pain: *“one to two times per week”* to *“most of the time”.*
- Pain interference with usual activities: *“sometimes”* to *“always”.*

To explore factors associated with pain intensity, frequency, and interference, cumulative link mixed modelling (CLMM) was used. Separate models were used to assess pain intensity, frequency of serious pain, and pain interference, respectively. Associations between biopsychosocial predictors and pain intensity, interference, and frequency of serious pain were examined using bivariate tests. Variables significantly associated with pain outcomes and those deemed particularly relevant to the study aims (i.e., rural-specific variables, including stoicism and pre-determinism, and variables known to be associated with pain, including age and comorbidities [44–46]) were included as covariates in their respective models, and crude and adjusted estimates were obtained.

**Persistent and intermittent pain.** Only participants who answered the pain intensity item of the AQoL-8D on at least four of the seven SAQs were included in this sub-analysis. Descriptive statistics (frequency, percentage) were used to describe the proportion of the sample who experienced no pain, intermittent pain, or persistent pain throughout the study period. examine factors associated with reporting “persistent” or “intermittent” pain (compared to “no pain”), binary logistic regression was used. Biopsychosocial predictors were included in their respective models if they were significantly associated with experiencing persistent or intermittent pain in bivariate tests.

## Results

A total of 659 rural cancer survivors completed at least one SAQ throughout the study period. Of these, 54% (n = 359) completed at least four SAQs, and 23% (n = 152) completed all seven SAQs. Participants were aged 64 years on average (*SD* = 11.3) and 47% were female. Participants typically resided in inner (47%) or outer (45%) regional areas and were in the most disadvantaged SEIFA tertile (57%). Most participants reported one comorbidity (68%). Of those with at least one comorbid health condition, most were considered “mild” risk (93%) as per CCI scoring criteria [34, 47]. The mean global psychological distress z-score was 1.50, indicating an elevated level of distress among the sample compared to population norms. Stoicism and predeterminism scores ranged from 1 to 5, with averages of 2.32 (*SD* = 0.9) and 2.67 (*SD* = 0.9), respectively. Characteristics of the sample at baseline are shown in Table 1.

**Table 1:** Baseline characteristics of rural cancer survivors included in the pain analysis (N = 659)

Characteristic		n (%)
Gender identity		
	Male	346 (53)
	Female	312 (47)
	Not disclosed	1 (0.002)
Remoteness		
	Inner regional	309 (47)
	Outer regional	295 (45)
	Remote/very remote	55 (8)
Socioeconomic status (tertile)		
	1 st (most disadvantaged)	378 (57)
	2nd	230 (35)
	3rd (least disadvantaged)	51 (8)
Cancer type		
	Breast	120 (18)
	Prostate	74 (11)
	Lung	47 (7)
	Colorectal	46 (7)
	Other	372 (57)
Recurrence status		
	Primary	513 (78)
	Recurrence	128 (19)
	Not specified	18 (3)
Time since diagnosis		
	< 1 year	424 (64)
	1 year—< 3 years	119 (18)
	3 years—< 5 years	42 (6)
	≥ 5 years	62 (9)
	Not specified	18 (3)
Number of comorbidities^a^		
	0	102 (15)
	1	447 (68)
	2 +	110 (17)
		Mean (SD)
Age (years)		64.4 (11.3)
Psychological distress^b^		1.50 (0.4)
Pre-determinism^c^		2.67 (0.9)
Stoicism^d^		2.32 (0.9)

^a^Charlson Comorbidity Index (CCI)

^b^Depression Anxiety and Stress Scale – 21 (DASS-21); presented as z score

^c^Pre-Determinism subscale of the Health Fatalism Scale; range: 1–5, higher scores indicate greater pre-determinism

^d^Stoicism subscale of the Mansfield Barriers to Help-Seeking Scale; range 1–5; higher scores indicate greater stoicism

### Descriptive pain outcomes

Over the study period (median 3 years follow-up), 84% of rural cancer survivors in the total sample experienced at least one episode of pain in the past week rated as *“moderate”, “severe”*, or *“unbearable”* intensity. The prevalence of *“severe”* intensity pain in the past week at individual timepoints ranged from 9 to 11% (see Table 2 for full pain prevalence results). Approximately 64% of the sample reported on at least one occasion that they experienced serious pain *“once per week”* or more, with the prevalence of serious pain experienced *“most of the time”* in the past week ranging from 12 to 15%. Almost two-thirds (66%) of rural cancer survivors reported on at least one occasion that pain had interfered with their usual activities *“sometimes”, “usually”, or “always”.* Across timepoints, 8% to 13% reported that pain *“usually”* interfered with typical activities over the past week.

**Table 2:** Factors associated with pain intensity, frequency of serious pain, and pain interference

		Pain intensity		Frequency of serious pain		Pain interference	
		OR (95%CI)	aOR (95%CI)	OR (95%CI)	aOR (95%CI)	OR (95%CI)	aOR (95%CI)
Age		0.99 (0.96, 1.01)	0.99 (0.98, 0.99)***	0.98 (0.96, 1.00)*	1.02 (1.01, 1.04) **	0.96 (0.95, 0.98)***	0.99 (0.97, 1.00)
Comorbidities							
	0	1.00	1.00	1.00	1.00	1.00	1.00
	1	0.99 (0.54, 1.82)	0.99 (0.46, 2.12)	0.90 (0.53, 1.54)	1.24 (0.76, 2.01)	0.75 (0.45, 1.24)	0.81 (0.50, 1.31)
	2 +	3.54 (1.67, 7.48)***	2.74 (1.11, 6.74)*	2.55 (1.32, 4.93)**	1.89 (1.05, 3.43) *	1.55 (0.84, 2.84)	1.59 (0.88, 2.87)
Cancer type – breast							
	Other	1.00	1.00	1.00	1.00	1.00	1.00
	Breast	0.37 (0.18, 0.79)**	0.40 (0.20, 0.83)*	-	-	0.66 (0.41, 1.10)	0.66 (0.42, 1.02)
Psychological distress		2.85 (2.31, 3.52)***	2.03 (1.54, 2.68) ***	2.55 (2.14, 3.05)***	1.40 (1.16, 1.67) ***	3.12 (3.09, 3.15)***	1.91 (1.59, 2.30)***
Pre-determinism		1.45 (1.14, 1.85)**	0.98 (0.71, 1.35)	1.32 (1.32, 1.33)***	1.01 (0.86, 1.30)	1.38 (1.11, 1.71)**	1.05 (0.86, 1.28)
Stoicism		1.45 (1.15, 1.84)**	1.08 (0.81, 1.45)	1.40 (1.14, 1.71)**	0.96 (0.80, 1.17)	1.41 (1.15, 1.72)**	0.97 (0.80, 1.16)

^*^p < 0.05, **p < 0.01, ***p < 0.001

A total of 395 rural caregivers provided measures of their pain intensity on at least four SAQs throughout the study period (60% of the analytic sample). Of these, 11% (n=44) consistently experienced *“no pain”* throughout the entire study period. Approximately 35% (n=140) experienced *“persistent”* pain, with the remaining 53% (n=211) experiencing *“intermittent”* pain.

### Pain intensity, frequency of serious pain, and pain interference

Full results of the bivariate analyses are presented in Supplementary File 2. At baseline, patients diagnosed with breast cancer had a moderate to large reduction in odds of pain intensity and interference scores (OR range: 0.35 to 0.61) compared to those with other cancer types. Higher psychological distress was associated with small to moderate increases in pain intensity, frequency of serious pain, and pain interference (OR range: 1.34 to 1.68). Reporting two or more comorbidities was also associated with a large increase in odds of frequent serious pain (OR = 2.09). Neither age, predeterminism, nor stoicism were significantly associated with baseline pain outcomes.

Full output of the CLMMs exploring factors associated with pain intensity, frequency of serious pain, and pain interference are displayed in Table 3. When controlling for all other sociodemographic, clinical, and psychological covariates, higher levels of psychological distress were associated with small to large increases in odds of higher pain intensity (aOR = 2.03, CI 1.54, 2.68), more frequent serious pain (aOR = 1.40, CI 1.16, 1.67), and greater interference in usual activities (aOR = 1.91, CI 1.59, 2.30). Younger age was also associated with a small increase in odds of frequent serious pain (aOR = 1.02, CI = 1.01, 1.04), but a small decrease in odds of pain intensity (aOR = 0.99, CI = 0.98, 0.99). Compared to those with no comorbid health conditions, those with two or more comorbidities had greater odds of reporting higher pain intensity (aOR = 2.74, CI, 1.11, 6.64) and more frequent serious pain (aOR = 1.89, CI 1.05, 3.43). Finally, being diagnosed with breast cancer (compared to other cancer types) was associated with a moderate decrease in odds of pain intensity (aOR = 0.40, CI 0.20, 0.83). Higher stoicism and predeterminism were associated with worse pain on all three outcomes in unadjusted analyses, but when accounting for covariates, these associations were no longer significant.

**Table 3:** Factors associated with odds of experiencing persistent or intermittent pain (versus no pain)

		Persistent Pain	Intermittent Pain		
		OR (95%CI)	aOR (95%CI)	OR (95%CI)	aOR (95%CI)
*Clinical factors*					
Comorbidities					
	0	1.00	1.00	-	-
	1	0.96 (0.81, 1.14)	0.95 (0.81, 1.12)	-	-
	2 +	1.22 (0.99, 1.49)	0.13 (0.94, 1.39)	-	-
*Psychological factors*					
Psychological distress		1.19 (1.12, 1.26) ***	1.18 (1.11, 1.25) ***	1.07 (1.01, 1.13) *	1.08 (1.02, 1.14) *
Pre-determinism		1.09 (1.02, 1.17) *	1.04 (0.97, 1.12)	1.02 (0.96, 1.07)	1.03 (0.97, 1.08)
Stoicism		1.03 (0.97, 1.10)	0.99 (0.97, 1.12)	0.99 (0.94, 1.05)	0.97 (0.92, 1.03)

^*^p < 0.05, **p < 0.01, ***p < 0.001

### Persistent and intermittent pain

Bivariate analyses revealed that psychological distress, comorbidities, and pre-determinism shared small to very large significant positive associations with persistent pain (*p* value range: *p* < 0.001 to *p* = 0.020), whereas only higher psychological distress was significantly associated with reporting intermittent pain (Cohen’s *d* = 0.38, *p* = 0.014). For full results of the bivariate analysis, see Supplementary File 2.

Full results of the binary logistic regression analyses are presented in Table 3. When including all covariates in the model, those with elevated levels of psychological distress had a small increase in odds of experiencing persistent (aOR = 1.18, CI 1.11, 1.25) and intermittent pain (aOR = 1.08, CI 1.02, 1.14), compared to those who experienced no pain throughout the study period. Crude analyses also showed that higher levels of predeterminism were associated with a small increase in odds of experiencing persistent pain (OR = 1.09, CI 1.02, 1.17) compared to no pain, but this effect was no longer significant when all covariates were controlled for.

## Discussion

Pain was commonly experienced by rural cancer survivors in our cohort, with at least four in five (84%) experiencing *“moderate”* to *“severe”* pain on at least one occasion throughout the study period. Similarly high rates of pain have been documented among cancer survivors in non-rural areas [48], suggesting that while the experience of pain is common among rural cancer survivors, it is not unique to this population group.

The longitudinal design of this study enabled the prevalence of persistent pain to be assessed, and this was experienced by 21% of rural cancer survivors in our sample. While this does not confer “chronic” pain, this study identifies for the first time that rural cancer survivors may be continually impacted by troublesome pain symptoms for many years after diagnosis and treatment. This highlights the importance of timely access to pain management and support in rural areas as ongoing, unmanaged pain can be detrimental to the physical and psychological wellbeing of cancer survivors [6, 8, 49, 50].

In line with the biopsychosocial model, our results reflect the influence of physical, social, and psychological factors on the experience of pain among cancer survivors. Individuals with more comorbidities reported elevated pain intensity and more frequent serious pain. This may arise because of the direct contribution of additional health conditions to pain, and because higher comorbidity burden is associated with increased risk of chronic pain [51]. In terms of social influences, we did not see a significant bivariate association between pain outcomes and participant gender, despite prior evidence that women are more likely to experience chronic pain [52]. Prior meta-analytic research has demonstrated a similar lack of sex differences in cancer pain [53], suggesting that other social factors may play a more significant role. Conversely, our results did indicate that younger cancer survivors had higher odds of more frequent and intense pain. Prior work has similarly shown that younger cancer survivors report worse pain intensity [54], and more frequent flares [55] compared to older cancer survivors, speculated to reflect differences in coping styles between generations [56, 57]. Older adults demonstrate lower distress at diagnosis [58] and report increased willingness to engage in coping strategies, such as seeking social support [59]. In contrast, younger adults may be less likely to engage in these coping strategies as work, social engagements, and leisure time, factors that typically comprise their personal identity, are likely to be impacted by cancer [60], exacerbating worry and distress. Furthermore, rural residents in this age group are more likely to engage in physically demanding occupations or assume multiple roles in their households and communities [61], making them vulnerable to the disruptive effects of pain. Together, these findings highlight the need for age- and context-sensitive pain management supports that are tailored to younger rural cancer survivors, to help sustain their wellbeing across the survivorship trajectory.

Among rural cancer survivors in our study, we saw psychological factors play a role in pain outcomes. Elevated psychological distress and was associated with worse pain outcomes, including greater odds of persistent pain. High distress levels may reflect the psychological consequences of unmanaged pain [6, 8], but distress itself can also amplify pain severity [62, 63]. For example, individuals with comorbid mental health conditions are at higher risk of developing chronic pain conditions, such as fibromyalgia [64]. With the measures of psychological distress (DASS-21) and pain outcomes (AQoL-8D) used in this study anchored to experiences within the past seven days, it is especially challenging to disentangle this potentially bidirectional association. Nevertheless, these findings highlight the compounding risk factors that can influence the experience of pain among rural cancer survivors and the importance of mental health support alongside pain management. This may be particularly pertinent in rural areas; those living in rural areas are more likely to report living with a mental health condition or other chronic illness, and less likely to report utilisation of chronic disease management services, compared to those in urban areas [65].

Stoicism and predeterminism are common elements of Australian rural culture, and may reflect the realities of living and working in harsher geographical and climatic conditions with fewer resources and opportunities for support [66]. In unadjusted analyses, participants in our study with higher stoicism and predeterminism reported higher pain intensity, more frequent serious pain, and greater pain interference, though when introducing covariates, these associations were attenuated. It is possible that the inclusion of other psychological processes – namely, psychological distress – may have masked or accounted for the impact of these attitudes on pain. This is especially possible given that predeterminism [67] and stoicism [68] are associated with greater anxiety and distress among cancer survivors. Nevertheless, we initially anticipated a negative association between stoic and predeterministic attitudes and pain, given their link to symptom under-reporting in medical contexts [14]. However, the non-confrontational nature of a survey may have encouraged more forthcoming reporting of pain in this population. The association between stoic, predeterministic attitudes, and worse pain may also reflect the impacts of ongoing avoidance of help-seeking or under-reporting of symptoms, leading to continually undermanaged and worse pain. Facilitating dialogue about attitudes towards discussing pain and normalising help-seeking may build rapport and facilitate open communication and access to support [14]. Additionally, interventions or models of care that consider the sociocultural factors that can contribute to under-reporting of pain could encourage earlier help-seeking for pain symptoms among rural cancer survivors. Rural generalists who are trained to provide comprehensive care across multiple specialty domains may be well suited to address the multidimensional nature of pain and facilitate access to pain support. However, ideal pain management involves multidisciplinary collaboration between primary care providers, allied health professionals, and specialists, and limited workforce availability and geographical isolation make such models difficult to implement in rural areas [69]. These challenges highlight the need for health systems to support rural clinicians in facilitating open conversations about pain and ensuring timely access to care.

### Strengths and limitations

This study was one of the first of its kind to explore pain outcomes in a large sample of rural cancer survivors, varying in cancer type and time since diagnosis; however, findings should be interpreted in context of some key limitations. Firstly, the measure of pain used in our study (AQoL-8D; [38] provides a generalised self-assessment of pain experienced in the last seven days. As such, it is unclear whether the pain reported by rural cancer survivors was acute, chronic, or cancer-related, Interpretation of these findings is further complicated by evidence that approximately 22% of rural Australians in the general (i.e., non-cancer) population experience chronic pain [11] Systematic review evidence suggests that pain measures implemented in cancer populations are often similarly non-specific [5]. With limited understanding of the exact type of presentation of pain experienced by rural cancer survivors, it is difficult to ascertain what types of pain management support are likely to be the most beneficial. Further research is needed to understand the factors that contribute to pain outcomes in this hard-to-reach population.

Furthermore, this study did not record information regarding participants’ stage of cancer at diagnosis or whether they received any form of physical, pharmacological, or psychological pain management support. Advanced cancer and failure to receive analgesic support may both contribute to worse pain outcomes [70, 71], however, we were unable to account for these factors as potential confounders in the observed pain outcomes. Results may have also been impacted by the inherently subjective nature of pain; what constitutes “serious” pain, for example, may differ between individuals and influence responding. Finally, attrition is common in longitudinal studies and may be a source of bias, especially due to its association between poorer quality of life and physical functioning [72, 73]. It is therefore possible that this study did not accurately capture the experiences of patients with particularly intense and interfering pain, who may have been more likely to opt-out of ongoing research participation.

## Conclusion

Pain is commonly experienced by rural cancer survivors, with younger adults and those with comorbid physical and mental health conditions most at risk. Higher psychological distress is associated with increased odds of persistent pain, but the direction of this association is unclear. Multidisciplinary supports targeting co-occurring health conditions may reduce risk of worsening pain in rural contexts, and cancer-specific pain measures are needed to disentangle the features of pain experienced among cancer survivors. With stoic and predeterministic attitudes also associated with worse pain, interventions that facilitate open discussions about pain and its management may be particularly relevant for rural communities.

## Supplementary Information

Below is the link to the electronic supplementary material.

Supplementary file1 (DOCX 31 KB)

Supplementary file2 (DOCX 25 KB)

## Data Availability

The data that support the findings of this study are not openly available due to reasons of sensitivity and are not available from the corresponding author.
