# Supplementary material for: Factors associated with pain among rural cancer survivors: Findings from an Australian longitudinal study
Source: Support Care Cancer. 2026 Mar 31;34(4):393. doi: 10.1007/s00520-026-10622-0 (PMC13038635; doi:10.1007/s00520-026-10622-0)
Supplement: Supplementary file 1 — Supplementary file1 (DOCX 31 KB) [file 520_2026_10622_MOESM1_ESM.docx]

**Supplementary File 1**

***Analytic Code for Data Analysis***

Nb. R version 4.4.0 was used for this analysis. “Pain function” herein refers to “pain interference”.

| **Analysis** | **Code** |
| --- | --- |
| Bivariate analyses to identify the factors associated with baseline pain intensity, frequency, and interference. | library(tidyverse)  library(purrr)  library(broom)  #For pain intensity  pain.int.biv.ordinal <- map_df(predictors, function(var) {    # Copy dataset  data_std <- full.data3    # Standardize numeric predictor only  if (is.numeric(data_std[[var]])) {  data_std[[var]] <- scale(data_std[[var]])[, 1] # Z-score  }    # Fit ordinal model  formula <- as.formula(paste("as.factor(pain_int_1)", "~", var))  model <- clm(formula, data = data_std)    # Tidy output and add odds ratio  broom::tidy(model) %>%  filter(term != "(Intercept)") %>%  mutate(  predictor = var,  odds_ratio = exp(estimate),  lower_ci = exp(estimate - 1.96 * std.error),  upper_ci = exp(estimate + 1.96 * std.error)  )  })  print(results.bivariate.ordinal.std, n = 60)  pain.freq.biv.ordinal <- map_df(predictors, function(var) {    # Copy dataset  data_std <- full.data3    # Standardize numeric predictor only  if (is.numeric(data_std[[var]])) {  data_std[[var]] <- scale(data_std[[var]])[, 1] # Z-score  }    # Fit ordinal model  formula <- as.formula(paste("as.factor(pain_freq_1)", "~", var))  model <- clm(formula, data = data_std)    # Tidy output and add odds ratio  broom::tidy(model) %>%  filter(term != "(Intercept)") %>%  mutate(  predictor = var,  odds_ratio = exp(estimate),  lower_ci = exp(estimate - 1.96 * std.error),  upper_ci = exp(estimate + 1.96 * std.error)  )  })  print(results.bivariate.ordinal.std, n = 60)  #Frequency  pain.freq.biv.ordinal <- map_df(predictors, function(var) {    # Copy dataset  data_std <- full.data3    # Standardize numeric predictor only  if (is.numeric(data_std[[var]])) {  data_std[[var]] <- scale(data_std[[var]])[, 1] # Z-score  }    # Fit ordinal model  formula <- as.formula(paste("as.factor(pain_freq_1)", "~", var))  model <- clm(formula, data = data_std)    # Tidy output and add odds ratio  broom::tidy(model) %>%  filter(term != "(Intercept)") %>%  mutate(  predictor = var,  odds_ratio = exp(estimate),  lower_ci = exp(estimate - 1.96 * std.error),  upper_ci = exp(estimate + 1.96 * std.error)  )  })  print(results.bivariate.ordinal.std, n = 60)  #Interference  pain.func.biv.ordinal <- map_df(predictors, function(var) {    # Copy dataset  data_std <- full.data3    # Standardize numeric predictor only  if (is.numeric(data_std[[var]])) {  data_std[[var]] <- scale(data_std[[var]])[, 1] # Z-score  }    # Fit ordinal model  formula <- as.formula(paste("as.factor(pain_func_1)", "~", var))  model <- clm(formula, data = data_std)    # Tidy output and add odds ratio  broom::tidy(model) %>%  filter(term != "(Intercept)") %>%  mutate(  predictor = var,  odds_ratio = exp(estimate),  lower_ci = exp(estimate - 1.96 * std.error),  upper_ci = exp(estimate + 1.96 * std.error)  )  })  print(results.bivariate.ordinal.std, n = 60) |
| Cumulative linear mixed modelling (CLMM) for pain intensity, frequency, and interference. | #Pivot longer  long.intensity <- full.data3 %>%  pivot_longer(cols = starts_with("pain_int_"),  names_to = "timepoint",  values_to = "pain.intensity")  glimpse(long.intensity)  library(ordinal)  #make sure pain.intensity is a factor  long.intensity$pain.intensity <- as.factor(long.intensity$pain.intensity)  #Run adjusted model with all covariates of interest first.  olmm.intensity.adj <- clmm(pain.intensity ~ age + cci.cat + breast.ca + dass_z + stoicism + fatalism + (1 \| id),  data = long.intensity)  summary(olmm.intensity.adj)  exp(coef(olmm.intensity.adj))  exp(confint(olmm.intensity.adj))  #Now crude ORs  #Age  olmm.intensity.age <- clmm(pain.intensity ~ age + (1\|id),  data = long.intensity)  summary(olmm.intensity.age)  exp(coef(olmm.intensity.age))  exp(confint(olmm.intensity.age))  #Breast ca  olmm.intensity.breast <- clmm(pain.intensity ~ breast.ca + (1\|id),  data = long.intensity)  summary(olmm.intensity.breast)  exp(coef(olmm.intensity.breast))  exp(confint(olmm.intensity.breast))  #CCI  olmm.intensity.cci <- clmm(pain.intensity ~ cci.cat + (1 \| id),  data = long.intensity)  summary(olmm.intensity.cci)  exp(coef(olmm.intensity.cci))  exp(confint(olmm.intensity.cci))  #DASS  olmm.intensity.dass <- clmm(pain.intensity ~ dass_z + (1 \| id),  data = long.intensity)  summary(olmm.intensity.dass)  exp(coef(olmm.intensity.dass))  exp(confint(olmm.intensity.dass))    #Fatalism  olmm.intensity.fatal <- clmm(pain.intensity ~ fatalism + (1 \| id),  data = long.intensity)  summary(olmm.intensity.fatal)  exp(coef(olmm.intensity.fatal))  exp(confint(olmm.intensity.fatal))  #Stoicism  olmm.intensity.stoic <- clmm(pain.intensity ~ stoicism + (1 \| id),  data = long.intensity)  summary(olmm.intensity.stoic)  exp(coef(olmm.intensity.stoic))  exp(confint(olmm.intensity.stoic))  #======= Frequency =============================  #Pain frequency  long.frequency <- full.data3 %>%  pivot_longer(cols = starts_with("pain_freq_"),  names_to = "timepoint",  values_to = "pain.frequency")  glimpse(long.frequency)  #make sure pain frequency is a factor  long.frequency$pain.frequency <- as.factor(long.frequency$pain.frequency)  #Run adjusted model with all covariates of interest first  olmm.frequency.adj <- clmm(pain.frequency ~ age + cci.cat + dass_z + stoicism + fatalism + (1 \| id),  data = long.frequency)  summary(olmm.frequency.adj)  exp(coef(olmm.frequency.adj))  exp(confint(olmm.frequency.adj))  #CCI  olmm.frequency.cci <- clmm(pain.frequency ~ cci.cat + (1 \| id),  data = long.frequency)  summary(olmm.frequency.cci)  exp(coef(olmm.frequency.cci))  exp(confint(olmm.frequency.cci))  #Age  olmm.frequency.age <- clmm(pain.frequency ~ age + (1 \| id),  data = long.frequency)  summary(olmm.frequency.age)  exp(coef(olmm.frequency.age))  exp(confint(olmm.frequency.age))  #DASS  olmm.frequency.dass <- clmm(pain.frequency ~ dass_z + (1 \| id),  data = long.frequency)  summary(olmm.frequency.dass)  exp(coef(olmm.frequency.dass))  exp(confint(olmm.frequency.dass))  #Stoicism  olmm.frequency.stoic <- clmm(pain.frequency ~ stoicism + (1 \| id),  data = long.frequency)  summary(olmm.frequency.stoic)  exp(coef(olmm.frequency.stoic))  exp(confint(olmm.frequency.stoic))  #Fatalism  olmm.frequency.fatal <- clmm(pain.frequency ~ fatalism + (1 \| id),  data = long.frequency)  summary(olmm.frequency.fatal)  exp(coef(olmm.frequency.fatal))  exp(confint(olmm.frequency.fatal))  #==== Pain function ======================  #Pain function  long.function <- full.data3 %>%  pivot_longer(cols = starts_with("pain_func_"),  names_to = "timepoint",  values_to = "pain.function")  #Make sure pain func is a factor  long.function$pain.function <- as.factor(long.function$pain.function)  #Run adjusted model with all covars of interest first  olmm.function.adj <- clmm(pain.function ~ age + breast.ca + cci.cat + dass_z + stoicism + fatalism + (1 \| id), data = long.function)  summary(olmm.function.adj)  exp(coef(olmm.function.adj))  exp(confint(olmm.function.adj))  #Crude ORs  #Age  olmm.function.age <- clmm(pain.function ~ age + (1\|id),  data = long.function)  summary(olmm.function.age)  exp(coef(olmm.function.age))  exp(confint(olmm.function.age))  #Breast  olmm.function.breast <- clmm(pain.function ~ breast.ca + (1\|id),  data = long.function)  summary(olmm.function.breast)  exp(coef(olmm.function.breast))  exp(confint(olmm.function.breast))  #CCI  olmm.function.cci <- clmm(pain.function ~ cci.cat + (1 \| id),  data = long.function)  summary(olmm.function.cci)  exp(coef(olmm.function.cci))  exp(confint(olmm.function.cci))  #Colorectal  olmm.function.colo <- clmm(pain.function ~ colorectal.ca + (1\|id),  data = long.function)  summary(olmm.function.colo)  exp(coef(olmm.function.colo))  exp(confint(olmm.function.colo))  #Prostate  olmm.function.pro <- clmm(pain.function ~ prostate.ca + (1\|id),  data = long.function)  summary(olmm.function.pro)  exp(coef(olmm.function.pro))  exp(confint(olmm.function.pro))  #DASS  olmm.function.dass <- clmm(pain.function ~ dass_z + (1\|id),  data = long.function)  summary(olmm.function.dass)  exp(coef(olmm.function.dass))  exp(confint(olmm.function.dass))  #Fatalism  olmm.function.fatal <- clmm(pain.function ~ fatalism + (1\|id),  data = long.function)  summary(olmm.function.fatal)  exp(coef(olmm.function.fatal))  exp(confint(olmm.function.fatal))  #Stoicism  olmm.function.stoic <- clmm(pain.function ~ stoicism + (1\|id),  data = long.function)  summary(olmm.function.stoic)  summary(olmm.function.stoic)  exp(coef(olmm.function.stoic))  exp(confint(olmm.function.stoic)) |
| Classifying persistent and intermittent pain | #First need to only include participants who have responded to at least 4 SAQs  full.data3.change <- full.data3 %>%  filter(rowSums(!is.na(select(., starts_with("pain_int_")))) >= 4)  #Need to identify those who have no pain, intermittent pain, persistent pain  #Convert to long format  full.data3.change.long <- full.data3.change %>%  pivot_longer(  cols = starts_with("pain_int_"),  names_to = "timepoint",  names_prefix = "pain_int_",  values_to = "pain_score"  ) %>%  mutate(timepoint = as.numeric(timepoint)) %>%  filter(!is.na(pain_score))  #Classify profiles  pain.summary <- full.data3.change.long %>%  group_by(id) %>%  summarise(  n_obs = n(),  n_zero = sum(pain_score == 0),  n_nonzero = sum(pain_score > 0),  all_zero = n_zero == n_obs,  all_non_zero = n_nonzero == n_obs,  .groups = "drop"  ) %>%  mutate(  pain.trajectory = case_when(  all_zero ~ "no pain",  all_non_zero ~ "persistent pain",  TRUE ~ "intermittent pain"  )  )  #Join back to main data  full.data3.change <- left_join(full.data3.change, pain.summary %>% select(id, pain.trajectory), by = "id")  #Descriptives - pain trajectory  table(pain.summary$pain.trajectory)  prop.table(table(pain.summary$pain.trajectory))*100 |
| Bivariate analyses to identify factors related to experiencing persistent and intermittent pain | #======== Bivariate analyses - what is assoc w persistent pain? ========  #Remove those with intermittent pain from this analysis - new DF  full.data3.change$pain.trajectory.x  full.data3.change <- full.data3.change %>%  select(-c(pain.trajectory.y))  full.data3.change <- full.data3.change %>%  rename("pain.trajectory" = "pain.trajectory.x")  full.data3.change.persist <- full.data3.change %>%  filter(pain.trajectory != "intermittent pain")  full.data3.change.persist$pain.trajectory <- as.factor(full.data3.change.persist$pain.trajectory)  #Age  ttest.age.persist <- t.test(age ~ pain.trajectory, data = full.data3.change.persist)  print(ttest.age.persist)    effectsize::cohens_d(age ~ pain.trajectory, data = full.data3.change.persist)  #Gender  table.gender.persist <- table(full.data3.change.persist$gender, full.data3.change.persist$pain.trajectory)  chisq.test(table.gender.persist)  effectsize::cramers_v(table.gender.persist)  #SEIFA  table.seifa.persist <- table(full.data3.change.persist$seifa_tertile, full.data3.change.persist$pain.trajectory)  chisq.test(table.seifa.persist)  table.seifa.persist  fisher.test(table.seifa.persist)  effectsize::cramers_v(table.seifa.persist)  #ARIA  table.aria.persist <- table.aria.persist[rownames(table.aria.persist) != "major city", ]  # Then create the table again  print(table.aria.persist)  # Now run chi-squared or Fisher's test  chisq.test(table.aria.persist)  fisher.test(table.aria.persist)  effectsize::cramers_v(table.aria.persist)  #Comorbidities  table.cci.persist <- table(full.data3.change.persist$pain.trajectory, full.data3.change.persist$cci.cat)  chisq.test(table.cci.persist)  effectsize::cramers_v(table.cci.persist)  #Cancer types  table.breast.persist <- table(full.data3.change.persist$pain.trajectory, full.data3.change.persist$breast.ca)  chisq.test(table.breast.persist)  effectsize::cramers_v(table.breast.persist)  table.colo.persist <- table(full.data3.change.persist$pain.trajectory, full.data3.change.persist$colorectal.ca)  chisq.test(table.colo.persist)  fisher.test(table.colo.persist)  effectsize::cramers_v(table.colo.persist)  table.prost.persist <- table(full.data3.change.persist$pain.trajectory, full.data3.change.persist$prostate.ca)  chisq.test(table.prost.persist)  effectsize::cramers_v(table.prost.persist)  table.lung.persist <- table(full.data3.change.persist$pain.trajectory, full.data3.change.persist$lung.ca)  chisq.test(table.lung.persist)  fisher.test(table.lung.persist)  effectsize::cramers_v(table.lung.persist)  #Recurrence  table.recur.persist <- table(full.data3.change.persist$pain.trajectory, full.data3.change.persist$recur.cat)  chisq.test(table.recur.persist)  effectsize::cramers_v(table.recur.persist)  #Time since dx  table.tsd.persist <- table(full.data3.change.persist$pain.trajectory, full.data3.change.persist$tsd)  chisq.test(table.tsd.persist)  fisher.test(table.tsd.persist)  effectsize::cramers_v(table.tsd.persist)  #Distress  tttest.dass.persist <- t.test(dass_z ~ pain.trajectory, data = full.data3.change.persist)  print(tttest.dass.persist)    effectsize::cohens_d(dass_z ~ pain.trajectory, data = full.data3.change.persist)  #Fatalism  ttest.fatal.persist <- t.test(fatalism ~ pain.trajectory, data = full.data3.change.persist)  print(ttest.fatal.persist)  effectsize::cohens_d(fatalism ~ pain.trajectory, data = full.data3.change.persist)  #Stoicism  ttest.stoic.persist <- t.test(stoicism ~ pain.trajectory, data = full.data3.change.persist)  print(ttest.stoic.persist)  effectsize::cohens_d(stoicism ~ pain.trajectory, data = full.data3.change.persist)  #==============Bivariate analyses - what is assoc w intermittent pain? ========  #Remove those with persistent pain from this analysis - new DF  full.data3.change.intermit <- full.data3.change %>%  filter(pain.trajectory != "persistent pain")  table(full.data3.change.intermit$pain.trajectory)  glimpse(full.data3.change)  glimpse(full.data3.change.intermit)  full.data3.change.intermit$pain.trajectory <- as.factor(full.data3.change.intermit$pain.trajectory)  #Age  ttest.age.inter <- t.test(age ~ pain.trajectory, data = full.data3.change.intermit)  print(ttest.age.inter)  effectsize::cohens_d(age ~ pain.trajectory, data = full.data3.change.intermit)  #Gender  table.gen.inter <- table(full.data3.change.intermit$pain.trajectory, full.data3.change.intermit$gender)  chisq.test(table.gen.inter)  effectsize::cramers_v(table.gen.inter)  #SEIFA  table.seifa.inter <- table(full.data3.change.intermit$pain.trajectory, full.data3.change.intermit$seifa_tertile)  chisq.test(table.seifa.inter)  fisher.test(table.seifa.inter)  effectsize::cramers_v(table.seifa.inter)  #ARIA  table.aria.inter <- table(full.data3.change.intermit$pain.trajectory, full.data3.change.intermit$aria)  chisq.test(table.aria.inter)  fisher.test(table.aria.inter)  table.aria.inter <- table.aria.inter[, colnames(table.aria.inter) != "major city"]  table.aria.inter  effectsize::cramers_v(table.aria.inter)  #Comorbidities  table.cci.inter <- table(full.data3.change.intermit$pain.trajectory, full.data3.change.intermit$cci.cat)  chisq.test(table.cci.inter)  fisher.test(table.cci.inter)  effectsize::cramers_v(table.cci.inter)  #Breast ca  table.breast.inter <- table(full.data3.change.intermit$pain.trajectory, full.data3.change.intermit$breast.ca)  chisq.test(table.breast.inter)  effectsize::cramers_v(table.breast.inter)  #Colorectal  table.colo.inter <- table(full.data3.change.intermit$pain.trajectory, full.data3.change.intermit$colorectal.ca)  chisq.test(table.colo.inter)  fisher.test(table.colo.inter)  effectsize::cramers_v(table.colo.inter)  #Prostate  table.prost.inter <- table(full.data3.change.intermit$pain.trajectory, full.data3.change.intermit$prostate.ca)  chisq.test(table.prost.inter)  effectsize::cramers_v(table.prost.inter)  #Lung  table.lung.inter <- table(full.data3.change.intermit$pain.trajectory, full.data3.change.intermit$lung.ca)  chisq.test(table.lung.inter)  fisher.test(table.lung.inter)  effectsize::cramers_v(table.lung.inter)  #Recurrence status  table.recur.inter <- table(full.data3.change.intermit$pain.trajectory, full.data3.change.intermit$recur.cat)  chisq.test(table.recur.inter)  effectsize::cramers_v(table.recur.inter)  #TSD  table.tsd.inter <- table(full.data3.change.intermit$pain.trajectory, full.data3.change.intermit$tsd)  chisq.test(table.tsd.inter)  fisher.test(table.tsd.inter)  effectsize::cramers_v(table.tsd.inter)  #DASS  full.data3.change.intermit$dass_z  ttest.dass.inter <- t.test(dass_z ~ pain.trajectory, data = full.data3.change.intermit)  print(ttest.dass.inter)  effectsize::cohens_d(dass_z ~ pain.trajectory, data = full.data3.change.intermit)  #Fatalism  ttest.fatal.inter <- t.test(fatalism ~ pain.trajectory, data = full.data3.change.intermit)  print(ttest.fatal.inter)  effectsize::cohens_d(fatalism ~ pain.trajectory, data = full.data3.change.intermit)  #Stoicism  ttest.stoic.inter <- t.test(stoicism ~ pain.trajectory, data = full.data3.change.intermit)  print(ttest.stoic.inter)  effectsize::cohens_d(stoicism ~ pain.trajectory, data = full.data3.change.intermit) |
| Binary logistic regression analyses to examine factors associated with experiencing persistent pain (versus no pain) and intermittent pain (versus no pain) | #Recode outcome as 0 (none) and 1 (persistent)  full.data3.change.persist <- full.data3.change.persist %>%  mutate(persistent = case_when(pain.trajectory == "no pain" ~ 0,  pain.trajectory == "persistent pain" ~ 1))  blr.model.persist <- glm(persistent ~ cci.cat + dass_z + stoicism + fatalism,  data = full.data3.change.persist)  summary(blr.model.persist)  exp(coef(blr.model.persist))  exp(confint(blr.model.persist))  #CCI  blr.persist.cci <- glm(persistent ~ cci.cat, data = full.data3.change.persist)  summary(blr.persist.cci)  exp(coef(blr.persist.cci))  exp(confint(blr.persist.cci))  #DASS  blr.persist.dass <- glm(persistent ~ dass_z, data = full.data3.change.persist)  summary(blr.persist.dass)  exp(coef(blr.persist.dass))  exp(confint(blr.persist.dass))  #Predeterminism  blr.persist.fatal <- glm(persistent ~ fatalism, data = full.data3.change.persist)  summary(blr.persist.fatal)  exp(coef(blr.persist.fatal))  exp(confint(blr.persist.fatal))  #Stoicism  blr.persist.stoic <- glm(persistent ~ stoicism, data = full.data3.change.persist)  summary(blr.persist.stoic)  exp(coef(blr.persist.stoic))  exp(confint(blr.persist.stoic))  #===== Binary logistic regression: intermittent ============  #Recode outcome as 0 (none) and 1 (intermittent)  full.data3.change.intermit <- full.data3.change.intermit %>%  mutate(intermittent = case_when(pain.trajectory == "no pain" ~ 0,  pain.trajectory == "intermittent pain" ~ 1))  blr.model.intermit <- glm(intermittent ~ dass_z + fatalism + stoicism,  data = full.data3.change.intermit)  summary(blr.model.intermit)  exp(coef(blr.model.intermit))  exp(confint(blr.model.intermit))  #Dass  blr.intermit.dass <- glm(intermittent ~ dass_z,  data = full.data3.change.intermit)  summary(blr.intermit.dass)  exp(coef(blr.intermit.dass))  exp(confint(blr.intermit.dass))  #Fatalism  blr.intermit.fatal <- glm(intermittent ~ fatalism,  data = full.data3.change.intermit)  summary(blr.intermit.fatal)  exp(coef(blr.intermit.fatal))  exp(confint(blr.intermit.fatal))  #Stoicism  blr.intermit.stoic <- glm(intermittent ~ stoicism,  data = full.data3.change.intermit)  summary(blr.intermit.stoic)  exp(coef(blr.intermit.stoic))  exp(confint(blr.intermit.stoic)) |
