# Supplementary material for: Factors associated with pain among rural cancer survivors: Findings from an Australian longitudinal study
Source: Support Care Cancer. 2026 Mar 31;34(4):393. doi: 10.1007/s00520-026-10622-0 (PMC13038635; doi:10.1007/s00520-026-10622-0)
Supplement: Supplementary file 2 — Supplementary file2 (DOCX 25 KB) [file 520_2026_10622_MOESM2_ESM.docx]

**Supplementary File 2**

**Bivariate predictors of baseline pain intensity, frequency and interference**

|  |  | **Pain intensity** | **Frequency of serious pain** | **Pain interference** |
| --- | --- | --- | --- | --- |
| ***Sociodemographic factors*** |  | \|  \| \| --- \|   **OR (95%CI)** | \|  \| \| --- \|   **OR (95%CI)** | \|  \| \| --- \|   **OR (95%CI)** |
| Age |  | 0.90 (0.70, 1.16) |  |  |
| Gender |  |  |  |  |
|  | Male | 1.00 | 1.00 | 1.00 |
|  | Female | 1.08 (0.65, 1.79) |  |  |
| Area-level socioeconomic status |  |  |  |  |
|  | 1^st^ quintile (most disadvantaged) | 1.00 | 1.00 | 1.00 |
|  | 2^nd^ quintile | 0.82 (0.47, 1.43) |  |  |
|  | 3^rd^ quintile (least disadvantaged) | 0.86 (0.34, 2.19) |  |  |
| Geographical remoteness |  |  |  |  |
|  | Inner regional | 1.00 | 1.00 | 1.00 |
|  | Outer regional | 1.23 (0.73. 2.08) |  |  |
|  | Remote/very remote | 0.58 (0.19, 1.75) |  |  |
| ***Clinical factors*** |  |  |  |  |
| Comorbidities |  |  |  |  |
|  | 0 | 1.00 | 1.00 | 1.00 |
|  | 1 | 1.29 (0.57, 2.89) |  |  |
|  | 2+ | 2.09 (0.86, 5.09) |  |  |
| Cancer type – breast |  |  |  |  |
|  | Other | 1.00 | 1.00 | 1.00 |
|  | Breast | **0.35 (0.15, 0.84) *** |  |  |
| Cancer type – colorectal |  |  |  |  |
|  | Other | 1.00 | 1.00 | 1.00 |
|  | Colorectal | 1.38 (0.57, 3.30) |  |  |
| Cancer type – prostate |  |  |  |  |
|  | Other | 1.00 | 1.00 | 1.00 |
|  | Prostate | 0.81 (0.33, 2.00) |  |  |
| Cancer type – Lung |  |  |  |  |
|  | Other | 1.00 | 1.00 | 1.00 |
|  | Lung | 1.17 (0.46, 2.94) |  |  |
| Cancer recurrence status |  |  |  |  |
|  | No | 1.00 | 1.00 | 1.00 |
|  | Yes | 1.53 (0.84, 2.78) |  |  |
| Time since cancer diagnosis |  |  |  |  |
|  | < 1 year | 1.00 | 1.00 | 1.00 |
|  | 1 - <3 years | 0.68 (0.33, 1.42) |  |  |
|  | 3 - <5 years | 0.51 (0.15, 1.74) |  |  |
|  | ≥ 5 years | 0.94 (0.41, 2.15) |  |  |
| ***Psychological factors*** |  |  |  |  |
| Psychological distress |  | **1.79 (1.43, 2.24) ***** |  |  |
| Pre-determinism^a^ |  | 1.14 (0.88, 1.47) |  |  |
| Stoicism^a^ |  | 1.27 (1.00, 1.61) |  |  |

p<0.05, **p<0.01, ***p<0.001

^a^ Predictors retained due to theoretical relevance.

**Bivariate associations between predictors and persistent / intermittent pain**

|  |  | **Persistent pain** | | **Intermittent pain** | |
| --- | --- | --- | --- | --- | --- |
| ***Sociodemographic factors*** |  | **Test statistic (df)^a^** | **Effect size (with measure)** | **Test statistic (df)^a^** | **Effect size (with measure)** |
| Age |  | 0.48 (74.2) | Cohen’s *d* = 0.08 | 0.29 (61.6) | Cohen’s *d* = 0.05 |
| Gender |  | 0.00 (1) | Cramer’s *V* = 0.00 | 0.10 (1) | Cramer’s *V* = 0.00 |
| Area-level socioeconomic status |  | NA | Cramer’s *V* = 0.00 | NA | Cramer’s *V* = 0.00 |
| Geographical remoteness |  | NA | Cramer’s *V* = 0.00 | NA | Cramer’s *V* = 0.00 |
| ***Clinical factors*** |  |  |  |  |  |
| Comorbidities |  | **8.96 (2) **** | Cramer’s *V* = 0.19 | NA | Cramer’s *V* = 0.03 |
| Breast cancer |  | <.01 (1) | Cramer’s *V* = 0.00 | <.01 (1) | Cramer’s *V* = 0.00 |
| Colorectal cancer |  | NA | Cramer’s *V* = 0.03 | NA | Cramer’s *V* = 0.04 |
| Prostate cancer |  | 0.44 (1) | Cramer’s *V* = 0.00 | <.01 (1) | Cramer’s *V* = 0.00 |
| Lung cancer |  | NA | Cramer’s *V* = 0.00 | NA | Cramer’s *V* = 0.00 |
| Cancer recurrence status |  | <.01 (1) | Cramer’s *V* = 0.00 | 0.05 (1) | Cramer’s *V* = 0.00 |
| Time since cancer diagnosis |  | NA | Cramer’s *V* = 0.07 | NA | Cramer’s *V* = 0.00 |
| ***Psychological factors*** |  |  |  |  |  |
| Psychological distress |  | **6.26 (88.6) ***** | Cohen’s *d* = 0.97 | **2.52 (68.6) **** | Cohen’s *d* = 0.38 |
| Pre-determinism^b^ |  | **2.37 (70.2) *** | Cohen’s *d* = 0.42 | 0.56 (61.0) | Cohen’s *d* = 0.10 |
| Stoicism^b^ |  | 1.08 (0.28) | Cohen’s *d* = 0.18 | 0.30 (63.0) | Cohen’s *d* = 0.05 |

^a^ *t* score for continuous variables, χ^2^ for categorical variables. Test statistic is NA where Fisher’s exact test was used.

^b^ Predictors retained due to theoretical relevance.

**p*<.05, ***p*<.01, ****p*<.001
